# A Scoping Review of Tools to Assess Diet in Children and Adolescents with Autism Spectrum Disorder

**DOI:** 10.3390/nu15173748

**Published:** 2023-08-27

**Authors:** Laura María Compañ-Gabucio, Carolina Ojeda-Belokon, Laura Torres-Collado, Manuela García-de-la-Hera

**Affiliations:** 1 Nutritional Epidemiology Unit (EPINUT), Department of Public Health, History of Science and Gynaecology, University of Miguel Hernández (UMH), 03550 Alicante, Spain; lcompan@umh.es (L.M.C.-G.); cojeda@umh.es (C.O.-B.); manoli@umh.es (M.G.-d.-l.-H.); 2 Alicante Institute for Health and Biomedical Research, ISABIAL, 03010 Alicante, Spain; 3 Spanish Consortium for Research on Epidemiology and Public Health (CIBERESP), Instituto de Salud Carlos III, 28034 Madrid, Spain

**Keywords:** neurodevelopmental disorders, childhood, questionnaires, diet, evaluation

## Abstract

Eating is considered one of the activities of daily living most affected by autism spectrum disorder (ASD) in children and adolescents and, therefore, needs to be thoroughly assessed using specific tools. The aim of this scoping review was to describe the most widely used tool to assess diet in children and adolescents with ASD. A search was conducted on PubMed, Scopus, EMBASE, Web of Science and PsycINFO databases. Two authors screened the articles and included all randomized or non-randomized studies published in English or Spanish in the last five years in which the diet of children and adolescents with ASD was assessed. Fifteen studies were included in this review. Mealtime behaviour was the most assessed variable in the included studies (*n* = 7). Thirteen different assessment tools were identified to evaluate the diet of children and adolescents with ASD, mainly at ages 2–12 (*n* = 11). The Brief Assessment scale for Mealtime Behavior in Children (BAMBI) and 24-h recalls were the most commonly used dietary assessment tools in the included studies. Our results can help professionals in the selection of an optimal scale to assess diet in children and adolescents with ASD.

## 1. Introduction

Autism spectrum disorder (ASD) is defined as a neurodevelopmental disorder mainly characterized by persistent deficits in social communication, difficulties in social interaction and a predisposition towards a pattern of stereotyped behaviours, interests and activities [1]. The etiological factors are diverse, and include epigenetic and environmental agents, although the definitive cause of ASD remains unknown [2,3]. These disorders manifest themselves at an early age, but are usually diagnosed later in childhood, at approximately 4 years of age [4]. According to the 2023 Centres for Disease Control and Prevention (CDC) report, the United States prevalence of ASD is 1 in 36 children, it is higher in boys than in girls and is continuously increasing [5,6].

Eating is one of the most affected daily life activities among children and adolescents with ASD, mainly because they tend to present hypersensitivity to some food textures, colours or tastes, which prevent them from trying new and unfamiliar foods [7,8]. This sensory alteration is usually manifested as repetitive, problematic and challenging behaviours at mealtimes, which may result in a restricted diet in terms of number and variety of foods [9,10,11]. This unvaried diet can even lead to nutrient inadequacy [12,13] and overweight [14,15], both of which can have negative effects on children’s and adolescents’ health [16]. Thus, there is an urgent need to develop dietary assessment tools for children and adolescents with ASD, which can be completed by parents and caregivers, to achieve a more accurate intervention in this issue.

There is evidence which supports that existing standardised methods for assessing feeding problems, eating behaviours and diet quality in ASD are very limited [17,18]. As dietary patterns are established in childhood and continue into adulthood [19], it is important to know and to use valid assessment tools to evaluate diet at an early age to identify the main nutrition necessities and to promote an optimal intervention. Currently, there are some published reviews on dietary assessment tools in children and adolescents with ASD. De Souza Silva et al. [20] carried out a Systematic Review (SR) aimed at evaluating the methodological quality of dietary assessment methods used to evaluate the diet intake of children and adolescents with ASD in clinical and epidemiological studies. This SR included eighty-nine articles and the results showed that most of them had a low-quality score. Holloway et al. [21] carried out a Scoping Review aimed at examining the evidence of validity and availability of measurement tools that evaluate usual dietary intakes and physical activity behaviours among individuals with ASD. In this review, one hundred and thirteen articles were included, and the results showed the need for more validated dietary tools in the ASD population. In this sense, we seek to perform a Scoping Review to complement the existent scientific evidence by answering the following research question: Which tool has been the most used to assess the diet of children and adolescents with ASD in intervention studies published in the last five years? The objective of this Scoping Review is to provide physicians and researchers with an updated source of information on the most widely used dietary assessment tool for children and adolescents with ASD.

## 2. Materials and Methods

We carried out a Scoping Review following the standards of the Cochrane Handbooks Version 6.2, 2021 [22] and the recommendations of the PRISMA Extension for Scoping Reviews (PRISMA-ScR) [23]. We conducted this type of review because our research question is broad and therefore could not have been addressed by a Systematic Review (SR). SRs are used to answer specific research questions which are usually related to the effectiveness, costs or effects of particular interventions [24,25]. We have not published a protocol of this review.

### 2.1. Search Strategy

On 10 August 2023, two of the authors of this review conducted a literature search in five databases: PubMed, Scopus, Web of Science, EMBASE and PsycINFO. We used the same search strategy and the same search terms in all these databases, using four different combinations: (1) (ASD OR autism OR autistic OR autistic OR asperger OR rett OR pervasive OR disintegrative), (2) (food OR diet), (3) (1 AND 2), (4) 1 AND 2 in the last 5 years. Time filtering (last 5 years) was used for all searches (Table 1). We applied this time filter because we wanted to provide a current synthesis of information. The time frame for considering an article to be current or not varies between disciplines, although in health and medical sciences it is recommended to use references from the last 5 years [26].

### 2.2. Review Criteria and Study Selection

We established for this review the following inclusion criteria: (1) articles published in English or Spanish; (2) articles available in full text; (3) articles whose study population consisted of persons ≤18 years old with Autism, Rett Syndrome, Asperger Syndrome, Disintegrative Disorder or Pervasive Developmental Disorder; (4) articles in which diet was evaluated; (5) articles with randomised or non-randomised study design. It should be noted that each intervention study has only been included once. In other words, publications derived from the same original intervention study were not included.

The process of study collection and subsequent data extraction was carried out independently by two authors (COB and LMCG), with a third author (LTC) intervening in case of discordance. In a Microsoft Excel spreadsheet, we downloaded all article titles retrieved from the different databases. On the Excel database, all studies were examined and selected for this scoping review by two authors using a four-stage screening process: elimination of duplicate articles, screening by title, screening by abstract and screening by full text. We then created the PRISMA Flow Diagram using the free-to-use web-based online tool available on the PRISMA website [27].

### 2.3. Data Extraction

In order to facilitate data extraction and avoid subjectivity on the part of the authors, we designed the items included in the tables a priori in accordance with the Cochrane Handbooks recommendations [22]. In one of the tables, we listed the general characteristics of the included studies as follows: author, year of publication, study design, study sample, country, participants, intervention/comparator, evaluation and dietary study outcomes. In another table, we listed the characteristics of the assessment tools used in the included studies as follows: dietary assessment tool used, author, year of publication, study participants, dietary assessment tool description, tool scores and assessment manager. Finally, we included a table containing items related to the risk of bias in the included studies, such as main limitations, funding sources and conflicts of interest.

### 2.4. Quality Assessment

We did not assess the quality of the studies included in this review, as it is not a mandatory requirement in scoping reviews [28]. Nevertheless, we have provided a table in which we have extracted and synthesised information related to the quality of the studies. In addition, we have described the main limitations of the included studies in the results section of this scoping review in considerable depth to allow readers to assess the results of the review in a more critical way.

## 3. Results

**Figure 1:**
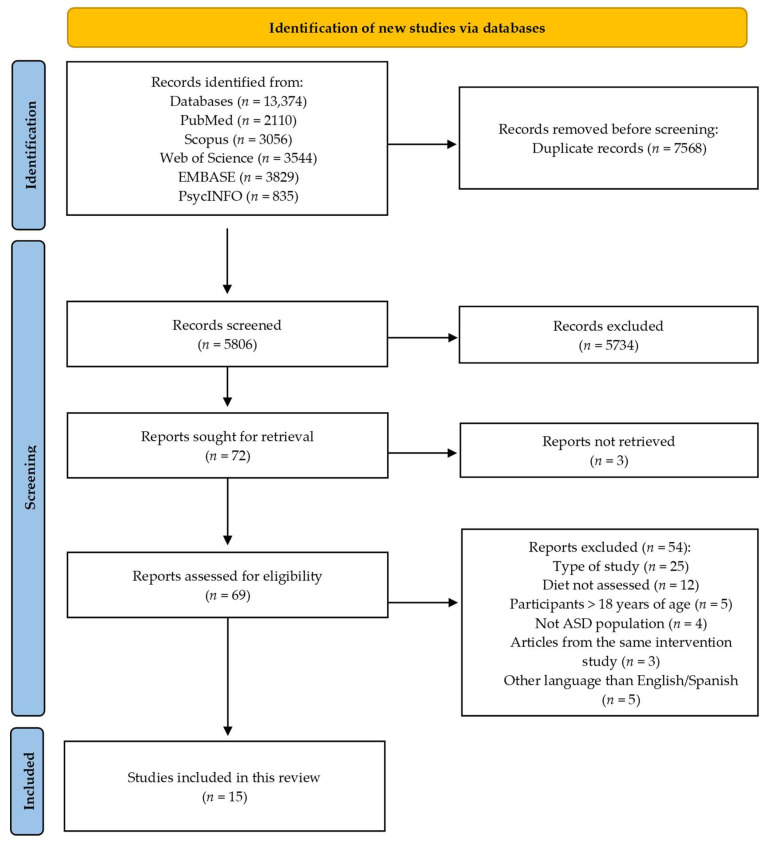
Flowchart of the study selection process.

The initial search strategy retrieved a total of 13,374 articles published in the last 5 years. After the removal of duplicate articles, 5806 articles remained for screening. During the screening, 4651 articles were discarded by title, 1083 articles by abstract and 54 articles by full text. In the full-text screening, three articles [29,30,31] were excluded as they were publications derived from previous intervention studies which were included [32,33]. At the end of this process 15 articles were included in this scoping review (Figure 1).

### 3.1. Main Characteristics of the Included Studies

Most of the studies (*n* = 4) were conducted in the United States [33,34,35,36]. The remaining studies were conducted in different countries such as Iran (*n* = 2) [37,38], Japan (*n* = 2) [39,40], Spain (*n* = 2) [41,42], United Kingdom (*n =* 1) [43], Poland (*n* = 1) [44], China (*n* = 1) [45], Iceland (*n* = 1) [32] and Korea (*n* = 1) [46] (Table 2). The year in which the most articles were published was 2019 (*n* = 4) [33,40,41,44], followed by 2020 (*n* = 3) [35,37,45], 2021 (*n* = 2) [32,38], 2017 (*n* = 2) [34,39], 2018 (*n* = 2) [43,46], 2022 (*n* = 1) [42] and 2023 (*n* = 1) [36]. Nine of the included studies were randomised clinical trials [32,33,36,37,38,41,42,44,46], and six [34,35,39,40,43,45] were a non-randomized clinical trial (Table 2).

The age of the participants in the included studies was between 2 and 12 years old (*n* = 11) [32,33,34,35,36,39,40,42,43,44,46], although in some studies participants of up to 17 years old [37,38,41,45] (*n* = 4) took part (Table 2). All participants had an ASD diagnosis, although in some studies children and adolescents with infantile cerebral palsy [34] as well as attention-deficit/hyperactive disorder (ADHD), anxiety and other neurodevelopmental disorders [32] also participated (Table 2). The ASD diagnosis was established by health professionals based on the International Statistical Classification of Diseases and Related Health Problems (ICD-10) [41,43,44], the Diagnostic and Statistical Manual of Mental Disorders, 4th Edition, Text Revision (DSM-IV-TR) [36,38,43] and the Diagnostic and Statistical Manual of Mental Disorders (DSM-5) [36,37,42,44,45] using different assessment tools, such as the Social Communication Questionnaire (SCQ) (*n* = 3) [33,39,44], the Autism Diagnostic Observation Scale (ADOS) (*n* = 3) [33,38,42] and the Childhood Autism Rating Scale (CARS) (*n* = 1) [37]. In some studies (*n* = 6) [32,34,35,36,40,46], the assessment tool used to confirm the ASD diagnosis was not stated.

### 3.2. Main Variables in the Included Studies

The most frequently studied variable in the included articles was mealtime behaviour (*n* = 7) [32,33,34,37,41,43,44], which included maladaptive and aggressive behaviours during mealtimes. The second most studied variables were food selectivity (*n* = 5) [35,38,39,43,45], which is defined as the number of foods that children and adolescents with ASD are able to tolerate from a range of foods [43] and dietary intake (*n* = 5) [36,37,38,42,46]. Mostly, studies outcomes were assessed before and after the intervention (*n* = 12) [32,34,35,36,38,39,40,42,43,44,45,46], although in a high proportion of included studies (*n* = 3) the assessment was also carried out during the intervention [33,37,41] (Table 2).

### 3.3. Dietary Assessment Tools and Questionnaires

Thirteen different dietary assessment tools were used in the included studies: the Brief Assessment scale for Mealtime Behavior in Children (BAMBI) or its revised version (BAMBI-R) (*n* = 4) [33,35,43,45], 24-*h* dietary recall (*n* = 4) [36,41,42,46], Food Frequency Questionnaires (FFQ) (*n* = 3) [38,42,45], 3-day food records (*n* = 2) [37,44], Dyadic Interaction Nomenclature for Eating (DINE) (*n* = 1) [35], Self-efficacy Assessment for Parents of Children with Selective Eating (SAPS) (*n* = 1) [39], Children’s Eating Behaviour Questionnaire (CEBQ) (*n* = 1) [32], Clinical Global Impression—Improvement Scale (CGI-I) (*n* = 1) [33], Children’s Eating Behavior Inventory (CEBI) (*n* = 1) [34], home eating records (*n* = 1) [40] and food indices (*n* = 1) [32] (Table 3). In two included studies, additional non-standardized questionnaires were used [39,43]. Health professionals usually used only one of these questionnaires to assess diet to interview parents and/or primary caregivers of children and adolescents with ASD, although in seven of the included studies more than one tool was used [32,33,35,39,42,43,45].

### 3.4. The BAMBI and the BAMBI-R

One of the most commonly used dietary assessment tool in the included studies was the BAMBI and its revised version the BAMBI-R (*n* = 4) [33,35,43,45] (Table 3). In the article by Galpin et al. [43], this tool was used to assess food selectivity in children and adolescents with ASD. Patton et al. [35] used the BAMBI to assess eating problems and eating behaviour in relation with the severity of ASD symptoms. Finally, Chung et al. [45] used this tool to assess how children and adolescents’ eating behaviour, diet and sensitivity may vary depending on the physical appearance of food.

In all these articles, the BAMBI was interpreted using the same scores, including a total score and three subscale scores. Questions can be answered with a Likert scale ranging from 1 (never/rarely) to 5 (at almost every meal) and a higher score represented a higher frequency of problematic behaviours and food selectivity. For its administration, health professionals asked the main caregivers to fill in the BAMBI based on observations during the mealtimes of children and adolescents with ASD.

The BAMBI-R is the revised version of the BAMBI, and their scores and interpretation are thus very similar. The main difference between these tools is that the BAMBI-R can be administered by teachers in the school setting. The BAMBI-R was less widely used (*n* = 1) [33] among included studies than the BAMBI (*n* = 3) [35,43,45], and it was used to evaluate the feasibility and effectiveness of a structured multidisciplinary intervention designed for children and adolescents with ASD with moderate food selectivity.

### 3.5. Twenty-four-h Dietary Recall

Another of the most commonly used dietary assessment tool in the included studies were 24-h dietary recalls (*n* = 4) [36,41,42,46] (Table 3). This dietary tool is a food diary that provides detailed information on portion sizes, preparation methods and quantities of food and beverages consumed by children in an entire day. In all the articles, the dietary recalls were filled out by parents. In the articles by Kim et al. [46], de la Torre-Aguilar et al. [42] and Kral et al. [36], three 24-h dietary recalls for two weekdays and a weekend day were used. In contrast, in the article by González-Domenech et al. [41] two 24-h dietary recalls per week were used. The type of analysis of 24-h dietary recalls was only specified in two articles. In the article by Kim et al. [46], the results were analysed using CAN-PRO 4.0, whereas in the article by Kral et al. [36], the University of Minnesota Nutrition Coordinating Center’s Food and Nutrient Database was used.

### 3.6. Main Limitations Reported in the Included Studies

The most reported limitation in the included studies was the small sample size (*n* = 9) [33,36,37,38,41,42,43,45,46], followed by the low generalisability of the results obtained (*n* = 4) [32,42,43,45], the absence of a control group (*n* = 3) [32,43,45] and the lack of blinding (*n* = 3) [33,35,44].

Limitations in relation to the type of questionnaires used or assessment performed were also reported in several articles. Some examples of this kind of limitations are the non-use of standardised questionnaires or the lack of comprehensive assessment of children’s selective eating and/or dietary variety (*n* = 3) [33,35,43], the use of questionnaires completed by parents (*n* = 1) [33], as well as the lack of direct observation by researchers (*n* = 1) [32].

Finally, limitations in relation to the intervention procedure were also reported, such as the participants’ difficulty in following the recommended diet (*n* = 3) [39,41,44], failure to correctly weigh or record children and adolescents’ food intake (*n* = 2) [32,45] or a short duration of the intervention (*n* = 1) [36] (Table 4).

## 4. Discussion

This scoping review aimed to identify the tools that have been used in experimental studies over the last five years to assess the diet of children and adolescents with ASD. Among the thirteen studies included in this Scoping Review, thirteen different dietary assessment tools were used: BAMBI, BAMBI-R, DINE, SAPS, FFQ, CEBQ, CGI-I, CEBI, 3-day food records, 24-h dietary recalls, home eating records, food indices and food questionnaires. The most commonly used tools were the BAMBI/BAMBI-R which was used to assess mealtime behaviour and food selectivity in children and adolescents with ASD and 24-h dietary recalls which were used to assess the dietary intake.

Most of the studies included in this review were conducted in the United States (*n =* 4). In this country, 1 in 36 children and adolescents have ASD [5,6], and 17% of this population suffers from obesity [47]. This high prevalence represents a significant health problem which is of great scientific interest. In fact, the Journal of the American Dietetic Association reflects that concerns about the adequacy of the diet of children and adolescents with ASD and the management of dietary selectivity are the main reasons for referral to nutrition services in the United States [48], which may partially justify a greater number of studies involving a wider variety of dietary assessment tools. The United States has a wide range of educational, medical, behavioural, nutritional and social services to identify cases with ASD and understand their needs and those of their families [49], which in some way may facilitate research on dietetics and nutrition in ASD.

In general, the dietary assessment tools used in the included studies were used to assess the diet of children and adolescents with ASD as a global concept, including sensory or behavioural aspects. In fact, mealtime behaviour was the most frequently assessed variable in the included articles. In this sense, different authors indicate that feeding difficulties in children and adolescents with ASD are mainly related to sensory and/or behavioural problems [50,51]. These problems may be manifested during mealtimes as playing with food, eating very slowly, filling their mouths with food, closing their mouths tightly, swallowing food without chewing or gagging continuously [10,50]. The high prevalence of these behaviours in children and adolescents with ASD [51] and their relationship with feeding can justify that the majority of the dietary assessment tools used include not only nutritional items but also behavioural and sensory ones.

The dietary assessment tools most commonly used in the included studies were the BAMBI/BAMBI-R and 24-h dietary recalls. On the one hand, one of the main arguments put forward by authors for the frequent use of the BAMBI is that it allows for the assessment of several areas affected in the feeding of children with ASD, such as food selectivity and behavioural characteristics of ASD related to sensory responses to food variability [35,43,45]. This can also be explained by the fact that this tool has been described as a one that addresses limitations existing in other tools, such as not measuring behavioural aspects in ASD which, as mentioned above, are very important in feeding [52]. In addition, the BAMBI presents good internal consistency, high validity and high test–retest reliability as well as a clear and solid structure for the measurement of the behaviours of this population [53]. Finally, a fact that may influence the more frequent use of this tool is its relatively easy administration, as it is completed by the parents of children and adolescents with ASD [52].

On the other hand, we have found a high use of 24-h dietary recalls to assess dietary intake in children and adolescents with ASD among included studies. Dietary intake is a very complex health behaviour, with large daily variations in the foods and beverages a person consumes, which makes its assessment complex. Twenty-four-h dietary recalls have been widely used in epidemiological studies to assess dietary intake due to its validity, high response rate, and simplicity [54]. This type of dietary tool is usually self-completed, but in the case of the paediatric population with or without ASD, 24-h dietary recalls are completed by parents or caregivers. This is because children are not familiar with the different methods of food preparation and do not have fully developed writing skills [55]. In this sense, a recently published review concluded that, although 24-h dietary recalls can underestimate energy intake, data collected using self-reported dietary assessment methods in children are highly valuable [56]. It should be noted that the collection of dietary information can be even more important and valuable in children and adolescents with ASD, as their communication limitations and sensory processing difficulties may affect diet, which can result in inadequate nutrient intake [16].

This scoping review has some limitations that need to be mentioned when interpreting our results. These results could be influenced by limitations common to most reviews, such as the lack of information reported in the included studies, publication bias, which limits null results of the interventions, and selection bias. We have possibly increased selection bias by only including articles published in the last five years, with full-text available and written in English or Spanish. In recent years, the classification of ASD has changed. Currently, it is considered a neurodevelopmental disorder in the DSM-5, and the specific disorders that were included in the ASD are no longer used. However, some recently published articles continue using these specific ASD. Thus, we decided to use the terms included in the DSM-IV definition (Autism, Rett Syndrome, Asperger Syndrome, Disintegrative Disorder or Pervasive Developmental Disorder) in our search strategy in order to not overlook some potential articles for our review. Finally, with regard to included studies, we need to point out that we have only included randomized and non-randomized clinical trials, which could contain biases related to this type of study design.

Our review also has some strengths. As far as we know, it is the first review that aims to describe the tools that have been used the most to assess the diet of children and adolescents with ASD in experimental studies. It is an up-to-date source of information as we have focused on research from the last 5 years. This review could be very useful for professionals involved in ASD treatment to select and use the dietary assessment that better fits their intervention objectives, as it provides a clear synthesis of different dietary assessment tools. Furthermore, this study has also identified some gaps in knowledge: the need for more studies to be carried out in Spain and at a European level and the need for studies with a larger sample size and with a greater post-intervention follow-up.

## 5. Conclusions

Two dietary assessment tools were the most widely used in children and adolescents with ASD. On the one hand, the BAMBI was widely used, possibly because it not only assesses diet but also other behavioural aspects that can alter different activities of daily living in this population. On the other hand, 24-h recalls were widely used, possibly because they assess a complex behaviour, namely dietary intake, in a simple parent-reported way. Dietary assessment tools in this population tend to provide information on food selectivity and mealtime behaviours, not only of the children and adolescents but also of their families and the environment in which mealtime takes place. The results of this scoping review could help different professionals assess diet in a more exhaustive and comprehensive manner, as well as promote the development and validation of more dietary assessment tools for children and adolescents with ASD.

## Figures and Tables

**Table 1:** Databases and search strategies used.

Databases	Search Strategy 10-August-2023	Results
PubMed		
#1	“arthropod struct dev” [Journal] OR “agron sustain dev” [Journal] OR “asd” [All Fields] OR (“autism s” [All Fields] OR “autisms” [All Fields] OR “autistic disorder” [MeSH Terms] OR (“autistic” [All Fields] AND “disorder” [All Fields]) OR “autistic disorder” [All Fields] OR “autism” [All Fields]) OR (“autistic disorder” [MeSH Terms] OR (“autistic” [All Fields] AND “disorder” [All Fields]) OR “autistic disorder” [All Fields] OR “autistic” [All Fields] OR “autistics” [All Fields] OR “autists” [All Fields]) OR (“asperger” [All Fields] OR “asperger s” [All Fields] OR “aspergers” [All Fields]) OR “Rett” [All Fields] OR (“pervasive” [All Fields] OR “pervasively” [All Fields] OR “pervasiveness” [All Fields]) OR “disintegrative” [All Fields]	109,644
#2	(“food” [MeSH Terms] OR “food” [All Fields] OR “diet” [MeSH Terms] OR “diet” [All Fields])	1,876,154
	#1 AND #2	4396
	#1 AND #2 in the last 5 years	2110
Scopus		
#1	TITLE-ABS-KEY ((asd OR autism OR autistic OR asperger OR rett OR pervasive OR disintegrative))	213,111
#2	TITLE-ABS-KEY ((food OR diet))	2,463,041
	#1 AND #2	6555
	#1 AND #2 in the last 5 years	3056
EMBASE		
#1	‘asd’/exp OR asd OR ‘autism’/exp OR autism OR autistic OR asperger OR rett OR pervasive OR disintegrative	178,684
#2	‘food’/exp OR food OR ‘diet’/exp OR diet	2,099,081
	#1 AND #2	7083
	#1 AND #2 in the last 5 years	3829
Web of Science		
#1	asd OR autism OR autistic OR asperger OR rett OR pervasive OR disintegrative (Topic)	209,781
#2	food OR diet (Topic)	2,679,024
	#1 AND #2	5994
	#1 AND #2 in the last 5 years	3544
PsycINFO		
#1	(ASD OR autism OR autistic OR asperger OR Rett OR pervasive OR disintegrative)	113,239
#2	(food OR diet)	136,365
	#1 AND #2	2076
	#1 AND #2 in the last 5 years	835

**Table 2:** Characteristics of the studies included in this scoping review.

Author, Year	Design	Sample (*n*), Country	Participants	Intervention/Comparator	Evaluation	Dietary Study Outcomes
Miyajima et al. [39], 2017	nRCT	49, Japan Loss to follow up (*n* = 26)	23 parents of children with ASD. Age between 3 and 6 years (mean age 4.40 years)	PEP on selective feeding/NA	2 months prior to the intervention, pre- and post- evaluation	Variety and number of foods consumed, and food selectivity
Taylor et al. [34], 2017	nRCT	58, United States Loss to follow up (*n* = 0)	58 children (25 with ASD, 33 with CP). Age between 1 and 12 years (mean age 5.8 years)	Intensive feeding program/NA	Pre-, and post-evaluation	Grams of food consumed, eating difficulties and mealtime behaviours
Galpin et al. [43], 2018	nRCT	23, United Kingdom Loss to follow up (*n* = 4)	19 children with ASD. Age between 4.5 and 10.5 years (mean age 6 years)	Sensory based selective eating intervention/NA	Pre- and post- evaluation	Food variety and mealtime behaviours
Kim et al. [46], 2018	RCT	42, Korea Loss to follow up (*n* = 7)	35 children with ASD. Age between 2 and 5.5 years (mean age 4.2 years)	Exposure program to vegetables/Usual treatment, applied behaviour analysis	Pre- and post- evaluation	Dietary intake
Sharp et al. [33], 2019	RCT	111, United States Loss to follow up (*n* = 73)	38 parent-child with ASD dyads. Age between 3 and 7 years (mean age 4.9 years)	MEAL/PEP	Pre-, at week 12 and post- evaluation. And 4 weeks after for intervention group.	Children’s eating problems improvement and mealtime behaviours.
Piwowarczyk et al. [44], 2019	RCT	79, Poland Loss to follow up (*n* = 13)	66 children with ASD. Age between 3 and 6 years (mean age 4 years).	Gluten-free diet/Gluten-containing diet	Pre- and post-evaluation	Adherence to the gluten-containing and to the gluten-free diet
González-Domenech et al. [41], 2019	RCT	40, Spain Loss to follow up (*n* = 11)	29 children with ASD. Age between 2 and 18 years (mean age 8.9 years).	Began with normal diet and ended with gluten-free and casein-free diet/Began with gluten-free and casein-free diet and ended with normal diet	Pre-, after first diet and after second diet evaluation	Adherence to the Diet Protocol
Yamane et al. [40], 2019	nRCT	40, Japan Loss to follow up (*n* = 0)	40 children with ASD. Age between 3 and 6 years (mean age NS).	Diet based on sensory factors/Diet based on visual appearance of foods/Diet based on familiar foods	Pre- and post-evaluation	Eating habits
Javadfar et al. [37], 2020	RCT	52, Iran Loss to follow up (*n* = 9)	43 children with ASD. Age between 3 and 13 years (mean age 8.9 years).	300–6000 IU/kg of Vitamin D/Placebo	Pre-, at week 8 and post- evaluation	Dietary intake
Chung et al. [45], 2020	nRCT	56, China Loss to follow up (*n* = 0)	56 children with ASD. Age between 8 and 15 years (mean age 10.7 years).	Exposure program to fruits and vegetables/NA	Pre- and post- evaluation	Fruits and vegetables acceptance, habitual fruits and vegetables consumption and mealtime behaviours
Patton et al. [35], 2020	nRCT	73, United States Loss to follow up (*n* = 0)	73 children with ASD. Age between 2 and 8 years (mean age 5.4 years).	Unfamiliar food presentation /NA	Pre- and post-evaluation	Mealtime behaviour
Doaei et al. [38], 2021	RCT	64, Iran Loss to follow up (*n* = 10)	54 children with ASD. Age between 5 and 15 years (mean age 8.2 years).	1000 mg omega-3/Placebo	Pre- and post-evaluation	Dietary intake
Thorsteinsdottir et al. [32], 2021	RCT	190, Iceland Loss to follow up (*n* = 109)	81 parent–child with ASD, ADHD or another ND dyads. Age between 8 and 12 years (mean age 10.4 years).	“Taste Education” program, immediate intervention/“Taste Education” program, delayed intervention	Pre-, post- and 6 months follow up evaluation	Fussy eating Food acceptance and variety
De la Torre-Aguilar et al. [42], 2022	RCT	117, Spain Loss to follow up (*n* = 9)	54 children with ASD. Age between 2 and 6 years (mean age 3.6 years).	800 mg omega-3 + 25 mg EPA/Placebo	Pre- and post-evaluation	Dietary intake and adequacy of food consumption
Kral et al. [36], 2023	RCT	38, United States Loss to follow up (*n* = 0)	38 parent–child with ASD dyads. Age between 6 and 10 years (mean age 8.6 years).	mHealth intervention/Education about healthy eating	Pre- and post-evaluation	Dietary intake

ADHD: attention-deficit/hyperactive disorder; ASD: autism spectrum disorder, CP: cerebral palsy; DHA: docosahexaenoic acid; EPA: Eicosapentanoic acid; GFD: gluten free diet; GD: gluten diet; GFCF: gluten free caffeine free; IU: International Units; MEAL: Food Aversion Management and Limited Variety; NA: Not applicable; ND: neurodevelopmental disorder (ASD or/and ADHD); NS: Not Stated; PEP: Parent Education Program; RCT: randomized controlled trial; nRCT: non-randomized controlled trial.

**Table 3:** Characteristics of the dietary assessment tools.

Dietary Assessment Tool Used	Author, Year	Participants and Diagnosis	Dietary Assessment Tool Description	Scores	Assessment Manager
BAMBI	Galpin et al. [43], 2018	19 children with ASD	18 items. A parent-reported standardized measure of mealtime behaviours. Three subscale scores were included: limited diversity, food refusal and features of autism.	Likert scale was used as scoring system ranging from 1 (Never/Rarely) to 5 (At almost every meal) for each question. Higher scores reflected more problematic mealtime behaviours.	Teachers, therapists and parents
	Chung et al. [45], 2020	56 children with ASD	18 items. A parent-reported standardized measure of mealtime behaviours with three subscale scores: limited variety, features of autism and food refusal.	Likert scale ranging from 1 (Never/Rarely) to 5 (always) was used for each question. Problematic mealtime behaviours were reflected by higher scores.	Not clearly stated
	Patton et al. [35], 2020	73 children with ASD	18 items. A parent-reported standardised measure of mealtime behaviours with three subscale scores: limited variety, features of autism and food refusal.	Scoring system based on a Likert scale ranging from 1 (Never/Rarely) to 5 (At Almost Every Meal) for each question. Higher scores reflected more problematic mealtime behaviours.	Study personnel
BAMBI-R	Sharp et al. [33], 2019	38 parent-child with ASD dyads	15 items. Questionnaire on mealtime behaviours common to children with ASD that contains four aspects (food selectivity, food refusal, mealtime rigidity disruptive and mealtime behaviours).	Five-point Likert scale measure each item, from 1 (never) to 5 (always). A total score ≥ 34 is considered clinically meaningful. Greater eating behaviour problems are reflected by higher scores.	Treatment assignment was performed by an independent evaluator.
24-h dietary recall	Kim et al. [46], 2018	25 children with ASD	Parent-reported food diary for three self-selected days (2 weekdays and 1 weekend day).	Dietary intakes were analysed via CAN-PRO 4.0, which provides the amount of intake across 60 nutrients.	Graduate students
	González-Domenech et al. [41], 2019	37 children with ASD	Unspecific number of items. Parents completed two 24-h recall per week which consisted of listing each food and beverage intake during the preceding 24 h.	Good compliant (adherence of 80–100% of the diet), intermediate compliant (adherence of 50–79%) and poor compliant (<50%).	Psychiatrist/ Psychologist
	De la Torre Aguilar et al. [42], 2022	54 children with ASD	Three non-consecutive 24-h dietary registrations. Parent-reported children’s following the Guidance on the Menu Methodology of the European Food Safety Agency	Not clearly stated.	Not clearly stated.
	Kral et al. [36], 2023	81 parent-child with ASD dyads	Three non-consecutive 24-h dietary registrations. Parent-reported questionnaires.	Calories consumption from salty and sugary snacks, sugar-sweetened beverages, water and fruits and vegetables were calculated using the University of Minnesota Nutrition Coordinating Center’s Food and Nutrient Database.	Dietitians
FFQ	Chung et al. [45], 2020	56 children with ASD	Number of items not stated. Pre- and post- caregiver-reported questionnaires were used to assess habitual fruits and vegetables consumption.	The frequencies of fruit and vegetables consumption were assessed using a five-point Likert scale ranging from 1 (never) to 5 (always).	Not clearly stated
	Doaei et al. [38], 2021	54 children with ASD	168 items. Parent-reported semi-quantitative validated tool to assess habitual food consumption.	Scores of questionnaires were transformed to grams/day using Iranian standard portions.	Nutritionist
	De la Torre-Aguilar et al. [42], 2022	54 children with ASD	FFQ: number of items not stated. Parent-reported measure to assess children’s dietary intake.	Not clearly stated.	Not clearly stated.
3-day food records	Piwowarczyk et al. [44], 2019	66 children with ASD	These records were obtained in two different moments of follow-up (week 2–4 and at week 12).	Adherence to the gluten-containing diet was described as the consumption in more than one meal every day of some gluten-containing foods. Adherence to the gluten-free diet was described as suitable when no intake of gluten was stated in the food record.	Study coordinators and psychologist
	Javadfar et al. [37], 2020	43 children with ASD	These records were collected at baseline, 8 and 15 weeks of the intervention.	Nutritionist IV software evaluated dietary intake. Energy and micro/macronutrients were calculated. Higher scores reflect higher intakes.	Dietitian
Food questionnaire	Miyajima et al. [39], 2017	23 parents of children with ASD	47 items. A non-standardized list of food which included carbohydrate-rich foods, liquids, meat, fish, beans, potatoes, seaweed, vegetables, mushrooms and eggs	Scoring system from 0 to 47 points. Higher scores represented higher food items consumed by children.	OT
	Galpin et al. [43], 2018	19 children with ASD	60 items. A non-standardized list which included 52 types of food, 3 liquids and 5 sauces.	Scoring system from 0 to 60 points. Higher scores represented higher variety of food consumed.	Teachers, therapists and parents
SAPS	Miyajima et al. [39], 2017	23 parents of children with ASD	12 items. It measures the degree of parental self-efficacy in three areas: rudimentary attitudes to eating, factors related to likes and dislikes and agreement to recommendations for selective eating.	Scoring system from 12 to 60 points. A Likert type scale was used; higher scores represented higher parental self-efficacy.	OT
CEBI	Taylor et al. [34], 2017	58 children (25 with ASD, 33 with CP).	40 items. Caregiver-report measure intended to assess eating and mealtime problems.	Two scores are obtained from the questionnaire: (i) the Total Eating Problems score which include a rate of 19 types of eating behaviours) and (ii) the Total Perceived Problems score (Behaviours that may or may not present a problem for the family). Higher scores represented higher eating and mealtime problems.	Feeding therapists
CGI-I	Sharp et al. [33], 2019	38 parent-child with ASD dyads	Unspecific number of items. Independent evaluator-rated, seven-point scale developed to measure the two most important feeding difficulties improvement.	Scores range from 1 (Very Much Improved) to 4 (Unchanged) to 7 (Very Much Worse). Very much improved or fairly improved (i.e., 2 or 1) were used to define the positive response; negative response was indicated by all other scores.	Treatment assignment was performed by an independent evaluator.
Household eating records	Yamane et al. [40], 2019	38 children with ASD	Records which included food preferences, environment and sensory tendencies.	Scores range from 1-5 (only milk; only carbohydrates; protein and carbohydrates; some vegetables; everything).	Nutritionist
DINE	Patton et al. [35], 2020	73 children with ASD	Unspecific number of items. This questionnaire included information about child’s eating and behaviour, and parent behaviour.	Family mealtime behaviours using the DINE were recorded and then videos were coded and analysed. This instrument does not have a final score.	Graduate students
CEBQ	Thorsteinsdottir et al. [32], 2021	81 parent-child with ASD, ADHD or another ND dyads	35 items. Parent-reported measure to assess children´s fussy eating.	Five-point Likert scale with 35 items, from “never” to “always”. Higher levels of fussy eating were indicated by a higher score for food fussiness. High levels of enjoyment were indicated by a high score for enjoyment of food.	Psychologist/ Nutritionist
Food indices	Thorsteinsdottir et al. [32], 2021	81 parent-child with ASD, ADHD or another ND dyads	57 items. Parent-reported intake of designated food items, clustered into three food indices (Fruit; nuts, seeds, and dried fruits).	Percentages of change in food acceptance and variety calculated with a dichotomous variable (accept or reject)	Psychologist/ Nutritionist

ADHD: attention-deficit/hyperactive disorder; ASD: autism spectrum disorder, BAMBI: brief assessment scale for mealtime behavior in children; BAMBI-R: revised version of the Brief Assessment scale for Mealtime Behavior in Children; CEBI: Children’s Eating Behavior Inventory; CEBQ: Children´s Eating Behaviour Questionnaire; CGI-I: Clinical Global Impression—Improvement Scale; CP: cerebral palsy; DINE: Dyadic Interaction Nomenclature for Eating; FFQ: food frequency questionnaire; ND: Neurodevelopmental disorders; OT: occupational therapy; SAPS: Self-efficacy Assessment for Parents of Children with Selective Eating.

**Table 4:** Risk of bias of the included studies.

Author, Year	Main Limitations	Funding/Support	Conflicts of Interest
Miyajima et al. [39], 2017	- A lack of an operational definition of selective eating. - No significant change in the degree of food items acceptable by children. - Loss of follow-up due to the difficulty of the parents to follow the recommendations. - Difficulties in addressing the subject in children who had solid selective eating.	No financial support of any kind.	None declared.
Taylor et al. [34], 2017	- Retrospective study. - The severity of CP and ASD diagnoses is unknown. - A lack of categorization based on the severity of motor impairment.	Not stated.	None declared.
Galpin et al. [43], 2018	- Small sample size. - Low generalizability of the results due to the heterogeneous group. - A lack of control group. - A lack of baseline control period. - A lack of meaningful standardized assessment measures. - Experimenter bias.	Not stated.	None declared.
Kim et al. [46], 2018	- Small sample size. - Convenient sampling method was used to select participants. - Large variability among participants because the different characteristics included in the feeding problems. - The observed variables did not meet normal distribution. - Non-significant changes in nutritional intake.	Not stated.	None declared.
Sharp et al. [33], 2019	- Small sample size. - Parent-completed questionnaires. - Interaction between food selectivity and disruptive behaviour. - A lack of standardized measures to assess dietary variety. - Changes in food selectivity severity were not assessed. - Treatment assignment was not blinded from parents.	Eunice Kennedy Shriver National Institute of Child Health and Human Development supported the study by grants to Emory University (MH081148).	None declared.
Piwowarczyk et al. [44], 2019	- Possible randomization bias due to some children possibly following a gluten free diet before the study. - Single blinding. - A lack of adherence to the allocated diet.	The Nutricia Foundation research Grant [RG8/2013] funded the study.	Some authors collaborate with Nutricia Foundation.
González-Domenech et al. [41], 2019	- Small sample size. - Difficulty for caregivers to follow recommendations. - Dietary errors outside the scope of the main caregiver. - Interindividual variability in relation to the age variable. - No conclusive results were found. - A lack of washing period between two interventions.	Not stated.	None declared.
Yamane et al. [40], 2019	- The support group classification was only based on observations.	Not stated.	None declared.
Javadfar et al. [37], 2020	- Small sample size. - The supplementation period was short.	Vice chancellor of research and technology of Kermanshah University of Medical Sciences funded the study as a thesis proposal for the MSc degree.	None declared.
Chung et al. [45], 2020	- Small sample size. - Low generalizability of the snack preparation to all type of foods. - A lack of control group. - A lack of statistical significance. - Only three fruits and three vegetables were studied.	No financial support of any kind.	None declared.
Patton et al. [35], 2020	- Families knew they were being observed during meals, fact that can reduce mealtime interactions. - Evaluators were not blinded. - Low internal consistency on some of the BAMBI subscales. - Specific questionnaires to assess sensory sensitivity were not used.	The Eunice Kennedy Shriver National Institute of Child Health and Human Development of the National Institutes of Health (R21HD076116); the Doctoral Student Research Award from the University of Kansas; and the Brown-Kirschmanv Award for Research Excellence from the University of Kansas supported the study.	None declared.
Doaei et al. [38], 2021	- Small sample size.	Not stated.	None declared.
Thorsteinsdottir et al. [32], 2021	- Low generalizability of the results due to not including children with ASD and lower-functioning. - No control comparison group with parental education sessions only. - No measurement of the weight of the food consumed by the children in the sessions was carried out. - Changes in children’s medications doses were not registered. - A lack of direct observation by researchers. - The sample had a high proportion of parents with higher education and full-time jobs, while there was a low proportion of single-parent homes.	The University of Iceland’s Research fund and the Public Health Fund of the Directorate of Health supported the study.	None declared.
De la Torre-Aguilar et al. [42], 2022	- Small sample size. - Loss of follow-up. - Low generalizability of the results due to the inclusion of only one centre. - Methods are different from other trials making it difficult to compare all their results.	Maternal-Infant and Developmental Health Network, Carlos III Health Institute	One author collaborated with Biosearch Life, a company that promoted the placebo and the nutritional supplement
Kral et al. [36], 2023	- Small sample size. - Some final evaluations could not be carried out due to COVID-19 pandemic restrictions. - Difficulties in enrolment due to COVID-19 pandemic restrictions. - Short duration of the intervention.	The Eunice Kennedy Shriver National Institute of Child Health and Human Development	One author had a financial conflict of interest related to the intellectual property of the mHealth nutrition intervention that was used in the study.

ASD: autism spectrum disorder, BAMBI: brief assessment scale for mealtime behavior in children; CP: cerebral palsy.

## Data Availability

The data presented in this study are available on request from the corresponding author.

## References

[1] American Psychiatric Association (2013). Diagnostic and Statistical Manual of Mental Disorders.

[2] Sauer A.K., Stanton J.E., Hans S., Grabrucker A.M., Grabrucker A.M. (2021). Autism Spectrum Disorders: Etiology and Pathology. Autism Spectrum Disorders.

[3] Taylor M.J., Rosenqvist M.A., Larsson H., Gillberg C., D’Onofrio B.M., Lichtenstein P., Lundström S. (2020). Etiology of Autism Spectrum Disorders and Autistic Traits over Time. JAMA Psychiatry.

[4] Choueiri R., Garrison W.T., Tokatli V. (2023). Early Identification of Autism Spectrum Disorder (ASD): Strategies for Use in Local Communities. Indian J. Pediatr..

[5] Shaw K.A., Bilder D.A., McArthur D., Williams A.R., Amoakohene E., Bakian A.V., Durkin M.S., Fitzgerald R.T., Furnier S.M., Hughes M.M. (2023). Early Identification of Autism Spectrum Disorder Among Children Aged 4 Years—Autism and Developmental Disabilities Monitoring Network, 11 Sites, United States, 2020. MMWR Surveill. Summ..

[6] Maenner M.J., Warren Z., Williams A.R., Amoakohene E., Bakian A.V., Bilder D.A., Durkin M.S., Fitzgerald R.T., Furnier S.M., Hughes M.M. (2023). Prevalence and Characteristics of Autism Spectrum Disorder Among Children Aged 8 Years—Autism and Developmental Disabilities Monitoring Network, 11 Sites, United States, 2020. MMWR Surveill. Summ..

[7] Patel M.D., Donovan S.M., Lee S.-Y. (2020). Considering Nature and Nurture in the Etiology and Prevention of Picky Eating: A Narrative Review. Nutrients.

[8] Van Dijk M.W.G., Buruma M.E., Blijd-Hoogewys E.M.A. (2021). Detecting Feeding Problems in Young Children with Autism Spectrum Disorder. J. Autism Dev. Disord..

[9] Sathe N., Andrews J.C., McPheeters M.L., Warren Z.E. (2017). Nutritional and Dietary Interventions for Autism Spectrum Disorder: A Systematic Review. Pediatrics.

[10] Saini V., Kadey H.J., Paszek K.J., Roane H.S. (2019). A Systematic Review of Functional Analysis in Pediatric Feeding Disorders. J. Appl. Behav Anal..

[11] Emond A., Emmett P., Steer C., Golding J. (2010). Feeding Symptoms, Dietary Patterns, and Growth in Young Children with Autism Spectrum Disorders. Pediatrics.

[12] Johnson C.R., Turner K., Stewart P.A., Schmidt B., Shui A., Macklin E., Reynolds A., James J., Johnson S.L., Manning Courtney P. (2014). Relationships between Feeding Problems, Behavioral Characteristics and Nutritional Quality in Children with ASD. J. Autism Dev. Disord..

[13] Suarez M.A., Nelson N.W., Curtis A.B. (2014). Longitudinal Follow-up of Factors Associated with Food Selectivity in Children with Autism Spectrum Disorders. Autism.

[14] Wallace G.L., Llewellyn C., Fildes A., Ronald A. (2018). Autism Spectrum Disorder and Food Neophobia: Clinical and Subclinical Links. Am. J. Clin. Nutr..

[15] Bandini L.G., Curtin C., Phillips S., Anderson S.E., Maslin M., Must A. (2017). Changes in Food Selectivity in Children with Autism Spectrum Disorder. J. Autism Dev. Disord..

[16] Esteban-Figuerola P., Canals J., Fernández-Cao J.C., Arija Val V. (2019). Differences in Food Consumption and Nutritional Intake between Children with Autism Spectrum Disorders and Typically Developing Children: A Meta-Analysis. Autism.

[17] Harris H.A., Mou Y., Dieleman G.C., Voortman T., Jansen P.W. (2022). Child Autistic Traits, Food Selectivity, and Diet Quality: A Population-Based Study. J. Nutr..

[18] Charman T., Gotham K. (2013). Measurement Issues: Screening and Diagnostic Instruments for Autism Spectrum Disorders—Lessons from Research and Practise. Child Adolesc. Ment. Health.

[19] Craigie A.M., Lake A.A., Kelly S.A., Adamson A.J., Mathers J.C. (2011). Tracking of Obesity-Related Behaviours from Childhood to Adulthood: A Systematic Review. Maturitas.

[20] De Souza Silva E., Castro K., Valle S.C., Dos Santos Vaz J. (2023). Dietary Assessment Methods Applied in Clinical and Epidemiological Studies in Children and Adolescents with Autism Spectrum Disorder: A Systematic Review. Rev. J. Autism Dev. Disord..

[21] Holloway J.M., Gray H.L., Buro A.W., Thomas J., Sauls R., Howard A.M. (2022). Measurement Tools to Assess Usual Dietary Intake and Physical Activity in Individuals with Autism Spectrum Disorder: A Scoping Review. Rev. J. Autism Dev. Disord..

[22] Higgins J.P.T., Thomas J., Chandler J., Cumpston M., Li T., Page M.J., Welch V.A. (2021). Cochrane Handbook for Systematic Reviews of Interventions Version 6.2 (updated February 2021).

[23] Tricco A.C., Lillie E., Zarin W., O’Brien K.K., Colquhoun H., Levac D., Moher D., Peters M.D.J., Horsley T., Weeks L. (2018). PRISMA Extension for Scoping Reviews (PRISMA-ScR): Checklist and Explanation. Ann. Intern. Med..

[24] Gough D., Thomas J., Oliver S. (2012). Clarifying Differences between Review Designs and Methods. Syst. Rev..

[25] Munn Z., Peters M.D.J., Stern C., Tufanaru C., McArthur A., Aromataris E. (2018). Systematic Review or Scoping Review? Guidance for Authors When Choosing between a Systematic or Scoping Review Approach. BMC Med. Res. Methodol..

[26] Santini A. (2018). The Importance of Referencing. J. Crit. Care Med..

[27] Page M.J., McKenzie J.E., Bossuyt P.M., Boutron I., Hoffmann T.C., Mulrow C.D., Shamseer L., Tetzlaff J.M., Akl E.A., Brennan S.E. (2021). The PRISMA 2020 Statement: An Updated Guideline for Reporting Systematic Reviews. BMJ.

[28] Arksey H., O’Malley L. (2005). Scoping Studies: Towards a Methodological Framework. Int. J. Soc. Res. Methodol..

[29] Thorsteinsdottir S., Njardvik U., Bjarnason R., Olafsdottir A.S. (2022). Changes in Eating Behaviors Following Taste Education Intervention: Focusing on Children with and without Neurodevelopmental Disorders and Their Families: A Randomized Controlled Trial. Nutrients.

[30] Thorsteinsdottir S., Bjarnason R., Eliasdottir H.G., Olafsdottir A.S. (2023). Body Composition in Fussy-Eating Children, with and without Neurodevelopmental Disorders, and Their Parents, Following a Taste Education Intervention. Nutrients.

[31] Burrell T.L., Scahill L., Nuhu N., Gillespie S., Sharp W. (2023). Exploration of Treatment Response in Parent Training for Children with Autism Spectrum Disorder and Moderate Food Selectivity. J. Autism Dev. Disord..

[32] Thorsteinsdottir S., Njardvik U., Bjarnason R., Haraldsson H., Olafsdottir A.S. (2021). Taste Education—A Food-Based Intervention in a School Setting, Focusing on Children with and without Neurodevelopmental Disorders and Their Families. A Randomized Controlled Trial. Appetite.

[33] Sharp W.G., Burrell T.L., Berry R.C., Stubbs K.H., McCracken C.E., Gillespie S.E., Scahill L. (2019). The Autism Managing Eating Aversions and Limited Variety Plan vs. Parent Education: A Randomized Clinical Trial. J. Pediatr..

[34] Taylor T., Kozlowski A.M., Girolami P.A. (2017). Comparing Behavioral Treatment of Feeding Difficulties and Tube Dependence in Children with Cerebral Palsy and Autism Spectrum Disorder. NeuroRehabilitation.

[35] Patton S.R., Odar Stough C., Pan T.Y., Holcomb L.O., Dreyer Gillette M.L. (2020). Associations between Autism Symptom Severity and Mealtime Behaviors in Young Children Presented with an Unfamiliar Food. Res. Dev. Disabil..

[36] Kral T.V.E., O’Malley L., Johnson K., Benvenuti T., Chittams J., Quinn R.J., Thomas J.G., Pinto-Martin J.A., Levy S.E., Kuschner E.S. (2023). Effects of a Mobile Health Nutrition Intervention on Dietary Intake in Children Who Have Autism Spectrum Disorder. Front. Pediatr..

[37] Javadfar Z., Abdollahzad H., Moludi J., Rezaeian S., Amirian H., Foroughi A.A., Nachvak S.M., Goharmehr N., Mostafai R. (2020). Effects of Vitamin D Supplementation on Core Symptoms, Serum Serotonin, and Interleukin-6 in Children with Autism Spectrum Disorders: A Randomized Clinical Trial. Nutrition.

[38] Doaei S., Bourbour F., Teymoori Z., Jafari F., Kalantari N., Abbas Torki S., Ashoori N., Nemat Gorgani S., Gholamalizadeh M. (2021). The Effect of Omega-3 Fatty Acids Supplementation on Social and Behavioral Disorders of Children with Autism: A Randomized Clinical Trial. Pediatr. Endocrinol. Diabetes Metab..

[39] Miyajima A., Tateyama K., Fuji S., Nakaoka K., Hirao K., Higaki K. (2017). Development of an Intervention Programme for Selective Eating in Children with Autism Spectrum Disorder. Hong Kong J. Occup. Ther..

[40] Yamane K., Fujii Y., Hijikata N. (2020). Support and Development of Autistic Children with Selective Eating Habits. Brain Dev..

[41] González-Domenech P.J., Díaz Atienza F., García Pablos C., Fernández Soto M.L., Martínez-Ortega J.M., Gutiérrez-Rojas L. (2020). Influence of a Combined Gluten-Free and Casein-Free Diet on Behavior Disorders in Children and Adolescents Diagnosed with Autism Spectrum Disorder: A 12-Month Follow-Up Clinical Trial. J. Autism Dev. Disord..

[42] de la Torre-Aguilar M.J., Gomez-Fernandez A., Flores-Rojas K., Martin-Borreguero P., Mesa M.D., Perez-Navero J.L., Olivares M., Gil A., Gil-Campos M. (2022). Docosahexaenoic and Eicosapentaenoic Intervention Modifies Plasma and Erythrocyte Omega-3 Fatty Acid Profiles But Not the Clinical Course of Children With Autism Spectrum Disorder: A Randomized Control Trial. Front. Nutr..

[43] Galpin J., Osman L., Paramore C. (2018). Sensory Snack Time: A School-Based Intervention Addressing Food Selectivity in Autistic Children. Front. Educ..

[44] Piwowarczyk A., Horvath A., Pisula E., Kawa R., Szajewska H. (2020). Gluten-Free Diet in Children with Autism Spectrum Disorders: A Randomized, Controlled, Single-Blinded Trial. J. Autism Dev. Disord..

[45] Chung L.M.Y., Law Q.P.S., Fong S.S.M. (2020). Using Physical Food Transformation to Enhance the Sensory Approval of Children with Autism Spectrum Disorders for Consuming Fruits and Vegetables. J. Altern. Complement. Med..

[46] Kim S.Y., Chung K.-M., Jung S. (2018). Effects of Repeated Food Exposure on Increasing Vegetable Consumption in Preschool Children with Autism Spectrum Disorder. Res. Autism Spectr. Disord..

[47] Sammels O., Karjalainen L., Dahlgren J., Wentz E. (2022). Autism Spectrum Disorder and Obesity in Children: A Systematic Review and Meta-Analysis. Obes. Facts.

[48] Cermak S.A., Curtin C., Bandini L.G. (2010). Food Selectivity and Sensory Sensitivity in Children with Autism Spectrum Disorders. J. Am. Diet. Assoc..

[49] Hyman S.L., Levy S.E., Myers S.M., Council On Children with Disabilities (2020). Section on Developmental and Behavioral Pediatrics Identification, Evaluation, and Management of Children with Autism Spectrum Disorder. Pediatrics.

[50] Malhi P., Saini S., Bharti B., Attri S., Sankhyan N. (2021). Sensory Processing Dysfunction and Mealtime Behavior Problems in Children With Autism. Indian Pediatr..

[51] Margari L., Marzulli L., Gabellone A., de Giambattista C. (2020). Eating and Mealtime Behaviors in Patients with Autism Spectrum Disorder: Current Perspectives. Neuropsychiatr. Dis. Treat..

[52] DeMand A., Johnson C., Foldes E. (2015). Psychometric Properties of the Brief Autism Mealtime Behaviors Inventory. J. Autism Dev. Disord..

[53] Lukens C.T., Linscheid T.R. (2008). Development and Validation of an Inventory to Assess Mealtime Behavior Problems in Children with Autism. J. Autism Dev. Disord..

[54] Salvador Castell G. (2015). ¿Qué y Cuánto Comemos? Método de Recuerdo 24 Horas. Nutr. Hosp..

[55] Foster E., Adamson A. (2014). Challenges Involved in Measuring Intake in Early Life: Focus on Methods. Proc. Nutr. Soc..

[56] Foster E., Bradley J. (2018). Methodological Considerations and Future Insights for 24-Hour Dietary Recall Assessment in Children. Nutr. Res..

